# Principles, Application, and Gaps of High-Intensity Ultrasound and High-Pressure Processing to Improve Meat Texture

**DOI:** 10.3390/foods12030476

**Published:** 2023-01-19

**Authors:** Yago Alves de Aguiar Bernardo, Denes Kaic Alves do Rosario, Carlos Adam Conte-Junior

**Affiliations:** 1 Graduate Program in Veterinary Hygiene (PPGHV), Faculty of Veterinary Medicine, Fluminense Federal University (UFF), Vital Brazil Filho, Niterói 24230-340, RJ, Brazil; 2 Center for Food Analysis (NAL), Technological Development Support Laboratory (LADETEC), Federal University of Rio de Janeiro (UFRJ), Cidade Universitária, Rio de Janeiro 21941-901, RJ, Brazil; 3 Laboratory of Advanced Analysis in Biochemistry and Molecular Biology (LAABBM), Department of Biochemistry, Federal University of Rio de Janeiro (UFRJ), Cidade Universitária, Rio de Janeiro 21941-909, RJ, Brazil; 4 Center for Agrarian Sciences and Engineering, Federal University of Espírito Santo (UFES), Alto Universitário, S/N Guararema, Alegre 29500-000, ES, Brazil

**Keywords:** muscles, emerging technologies, beef industry, tenderness, cavitation, connective tissue

## Abstract

In this study, we evaluate the most recently applied emerging non-thermal technologies (NTT) to improve meat tenderization, high-intensity ultrasound (HIUS), and high-pressure processing (HPP), aiming to understand if individual effects are beneficial and how extrinsic and intrinsic factors influence meat toughness. We performed a systematic literature search and meta-analysis in four databases (Web of Science, Scopus, Embase, and PubMed). Among the recovered articles (*n* = 192), 59 studies were included. We found better sonication time in the range of 2–20 min. Muscle composition significantly influences HIUS effects, being type IIb fiber muscles more difficult to tenderize (*p* < 0.05). HPP effects are beneficial to tenderization at 200–250 MPa and 15–20 min, being lower and higher conditions considered inconclusive, tending to tenderization. Despite these results, undesirable physicochemical, microstructural, and sensory alterations are still unknown or represent barriers against applying NTT at the industrial level. Optimization studies and more robust analyses are suggested to enable its future implementation. Moreover, combining NTT with plant enzymes demonstrates an interesting alternative to improve the tenderization effect caused by NTT. Therefore, HIUS and HPP are promising technologies for tenderization and should be optimized considering time, intensity, pressure, muscle composition, undesirable changes, and combination with other methods.

## 1. Introduction

Meat texture is a fundamental aspect of the sensory evaluation of consumers. The development of an acceptable texture is directly related to meat processing [1]. After slaughter, musculature undergoes a series of biochemical changes, including reducing oxygen (O_2_) levels and using the glycolytic pathway to obtain the adenosine triphosphate (ATP) necessary for metabolism [2]. The release of lactic acid resulting from glycolysis is responsible for providing an acidic environment, allowing the activity of proteolytic enzymes, such as the calpains, which contribute to the progressive tenderization of meat during storage [3]. This phenomenon of muscle transforming into meat and later maturation is known as aging [4]. During this process, tenderness and water-holding capacity are improved, and several flavor-related metabolites, such as leucine and isoleucine, contribute to meat palatability [5]. However, aging is time-consuming, and obtaining a product with acceptable textural properties can take at least six to seven days for beef [6,7], and is also related to some undesirable changes, such as color instability and discoloration [8,9]. Moreover, as recently reviewed by Warner et al. [10], an extensive range of pre-slaughter (*antemortem*) and post-slaughter (*postmortem*) factors could affect meat tenderization. In this context, the pre-slaughter factors, such as (i) breed and genotype, (ii) presence of mutant genes (e.g., myostatin), (iii) use of growth promotants, (iv) animal age, (v) castration, and (vi) type of feeding system, represent, at some extent, challenges to overcome to improve tenderness, as they interact with metabolic, molecular, and enzymatic processes directly related to meat tenderization [10]. Therefore, there is a need to promote improvements to optimize the aging process, such as using novel promising meat tenderizing methods, such as non-thermal technologies (NTT).

Recently, Bhat et al. [11] published a narrative review regarding NTT application to meat tenderization, highlighting high-intensity ultrasound (HIUS) and high-pressure processing (HPP). Rosario et al. [12] stated that these two NTTs have attracted consumers’ attention due to the reduction of water and energy consumption during food processing. The use of HIUS and HPP as meat tenderizing technologies has shown an increase in the last two decades, being elegantly discussed by reviews and research papers [13,14,15,16,17]. Nevertheless, these studies are perspectives that have not deeply and statistically addressed the impacts and technological barriers of HIUS and HPP on meat tenderization. Despite the promising effects of these technologies, there is still no consensus about the suitability of its application to meat tenderization (Table 1 and Table 2), as well as the optimal parameters (HIUS time and intensity, HPP time and pressure), and the possible influences of the structural composition of the treated muscle.

Therefore, this study aimed to (i) evaluate the use of HIUS and HPP to promote the tenderization of meat, (ii) understand how intrinsic (muscle composition) and extrinsic factors can influence the effects of HIUS and HPP, (iii) describe other matrix changes associated with NTT processing during tenderization, and (iv) address its practical application.

## 2. Materials and Methods

### 2.1. Protocol of the Systematic Data Search

This paper was performed based on the P (population), I (intervention), C (comparison), and O (outcome) methodology. Therefore, the focus question was if it is possible to use the HIUS and HPP (intervention) in muscle samples (population) to reach improved tenderization based on shear force values (outcome) in comparison with non-treated samples. Moreover, the study’s key objective was to revise and analyze the applicability of HIUS and HPP to meat tenderization.

### 2.2. Outcomes of Interest

#### 2.2.1. Primary Outcome

The primary outcome of interest was to assess non-thermal methods (HIUS and HPP) as tenderization technologies for the meat industry by evaluating and comparing the shear force values for control and treated samples.

#### 2.2.2. Secondary Outcomes

Determine the effects of extrinsic factors (e.g., processing time, intensity, pressure) and intrinsic factors (fiber type) on the resulting shear force statistically and correlate these variables to find optimal ranges for the application.

Describe and discuss physicochemical, microstructural, and sensory alterations associated with meat tenderization after NTT application and using papain as an adjuvant.

Address scientific gaps and further studies focusing on meat tenderization assisted by HIUS and HPP to overcome barriers to its implementation.

### 2.3. Inclusion and Exclusion Criteria

#### 2.3.1. Inclusion Criteria

The inclusion criteria adopted in this study were: (i) the use of bovine or pork raw meat (muscle) as the matrix, detailed by muscle name, (ii) the specification of treatments conditions, such as the time (min), intensity (W/cm^2^), equipment type, pressure (MPa), temperature (°C), depending on the NTT, (iii) the measurement of meat texture through shear force (Newton), and (iv) full peer-reviewed papers, in English.

#### 2.3.2. Exclusion Criteria

The exclusion criteria included: (i) studies using chilled meat, frozen meat, or meat batter as the matrix, not raw meat itself, (ii) papers that investigated the use of the NTT as a processing, such as thawing, extraction, marinating, or drying technology, leading to changes in the methodology and matrix composition, (iii) studies where muscles were processed before treatment (e.g., electrically stimulated), (iv) studies without mean and/or standard deviation, and the number of samples (*n*) for the shear force obtained after the treatment, given by text, table or figure data, (v) samples not treated inside vacuum-packaged bags, (vi) literature reviews and meta-analysis, and (vii) duplicate or triplicate research articles.

### 2.4. Search Strategy

A data survey was performed in February 2022, given published studies on applying the selected NTTs for meat tenderization. In this study, four databases were consulted to access the data: (i) Web of Science, (ii) Scopus, (iii) PubMed, and (iv) Embase. The search retrieved articles according to the search terms (ST) adopted to attend the PICO strategy. The terms were chosen to cover the maximum number of articles available in these databases whose population was composed of the main muscle groups used for meat production and consumption, the use of NTT as an intervention, and the response provided through the shear force values.

Search term 1 (ST1): “longissimus” OR “semimembranosus” OR “semitendinosus” OR “infraspinatus” OR “cleidoocciptalis” (population).

Search term 2 (ST2): “ultrasound” OR “sonication” OR “high pressure” OR “hydrostatic pressure” OR “HHP” OR “HPP” (intervention).

Search term 3 (ST3): “shear force” OR “tenderization” (outcome).

ST1 was established according to previous searches to verify the main HIUS-treated muscles reported in the literature. *Longissimus* was identified as the most used muscle for tenderizing studies by sonication. The first search was performed using only *longissimus* as ST1. According to the first papers obtained, the remaining identified muscles were added to ST1 so that the effects of muscle composition became a variable to be analyzed by this study. The ST2 (intervention) was established considering the equipment employed (high-intensity ultrasound and high-pressure processing). ST3 represents the desired response for the treated muscles, the tenderization, measured by the shear force.

After determining the search components, the Boolean operator “AND” was employed to combine the terms and compose the final search string [(“longissimus” OR “semimembranosus” OR “semitendinosus” OR “infraspinatus” OR “cleidoocciptalis”) AND (“ultrasound” OR “sonication” OR “high pressure” OR “hydrostatic pressure” OR “HHP” OR “HPP”) AND (“shear force” OR “tenderization”)]. The results were reported according to the Preferred Reporting Items for Systematic Review and Meta-Analyses statement (PRISMA) [31]. Thus, the duplicated or triplicated identified references were eliminated. The remaining papers were selected according to title and abstract, excluding reviews, systematic reviews, and meta-analyses. After selection, the manuscripts were read in full, and the inclusion and exclusion criteria were used to select the articles to include in this study.

### 2.5. Studies Quality Evaluation

The included articles were subjected to a quality evaluation step using the Standard Quality Assessment Criteria for Evaluating Primary Research Papers from a Variety of Fields [32] to reduce the possible risks of bias in this meta-analysis.

### 2.6. Risk of Bias Assessment

Possible sources of bias in this meta-analysis include (i) language, (ii) the small number of articles published and included, (iii) chosen databases, (iv) inclusion and exclusion criteria, and (v) the impact of missing data.

### 2.7. Data Extraction and Statistical Analysis

The data extraction included: author names, year of publication, sampling size (*n*), and shear force values (mean and standard deviation). For articles whose dependent variable (shear force) was given in kilogram-force (kgf), a correction factor (Equation (1)) was applied to standardize the unit in Newton (N):

(1) $$Shearforce_{N}=Shearforce_{kgf}\times 9.8$$

The forest plots were performed to measure the effects of meat sonication under different conditions (independent variable). The influence of the non-thermal technologies on meat tenderization was evaluated according to (i) the extrinsic treatment conditions and (ii) the intrinsic composition of the treated muscle (*Longissimus thoracis et lumborum* (*L. dorsi*)*, L. thoracis, L. lumborum, Semimembranosus, Semitendinosus, Infraspinatus,* and *Cleidoocciptalis*). Thus, statistical analyses were performed by each one of these subgroups, generating a forest plot for each independent variable. Moreover, the reduction or increase in the shear force average using a 95% confidence interval (CI) was measured for each subgroup.

Statistical analyses were performed in *Review Manager^©^* software (RevMan version 5.4, Nordic Cochrane Center, Copenhagen, Denmark), according to its algorithms [33]. The pooled shear force rates were determined using each study’s means and standard deviations (obtained from the included articles’ tables, figures, and texts). Due to the substantial heterogeneity between the included studies, we applied a random effect model using the inverse-variance method [34]. Results were plotted, including the standardized mean difference (SMD), the effect size, and the weight of each study. The general effect size for each analyzed subgroup was represented graphically by the diamond in the forest plots. Results to the left of the central line of the graph favor the use of non-thermal technology as a tenderizing technology (*p* < 0.05), and results to the right favor untreated sample tenderness (*p* < 0.05). Results that crossed the central line were considered inconclusive (*p* > 0.05).

The individual weight of each study was measured according to the inverse-variance method, as the squared standard error provided by the study, as shown in Equation (2):

(2) $$w_{i}=\frac{1}{{\left(SE\;\left\{\theta_{i}\right\}\right)}^{2}}$$

where: *w_i_* is the weight of an individual study, and *SE* (*θ_i_*) is the standard error of an individual intervention effect estimate.

Using *w_i_*, it was possible to calculate the inverse-variance effect estimate using Equation (3):

(3) $$\theta_{IV}=\frac{\sum w_{i}\;\cdot \;\theta_{i}}{\sum w_{i}}$$

where: *θ_IV_* is the inverse-variance effect estimate, and *θ_i_* is an individual intervention effect estimate.

The heterogeneity among the included studies was checked by the inverse-variance method through Higgins and Thompson’s *I*^2^ test (or *I*^2^-statistic). The result could be interpreted as the proportion of the total variation in the study estimate due to heterogeneity [33,35]. To access *I*^2^, the chi-square (Q*_IV_*) statistic was first calculated, according to Equation (4):

(4) $$Q_{IV}=\sum w_{i}{\left(\theta_{i}-\theta_{IV}\right)}^{2}$$

*I*^2^ limits were assessed considering the following levels of heterogeneity (i) low (0–40%), (ii) moderate (30–60%), (iii) substantial (50–90%), and (iv) considerable (75–100%), and was calculated by the software as:

(5) $$I^{2}\left(\%\right)=\frac{Q_{IV}-df}{Q_{IV}}\times 100$$

where: *df* is the degree of freedom (number of studies that composes the group or subgroup minus 1).

## 3. Results and Discussion

### 3.1. Data Search and Studies Characteristics

**Figure 1:**
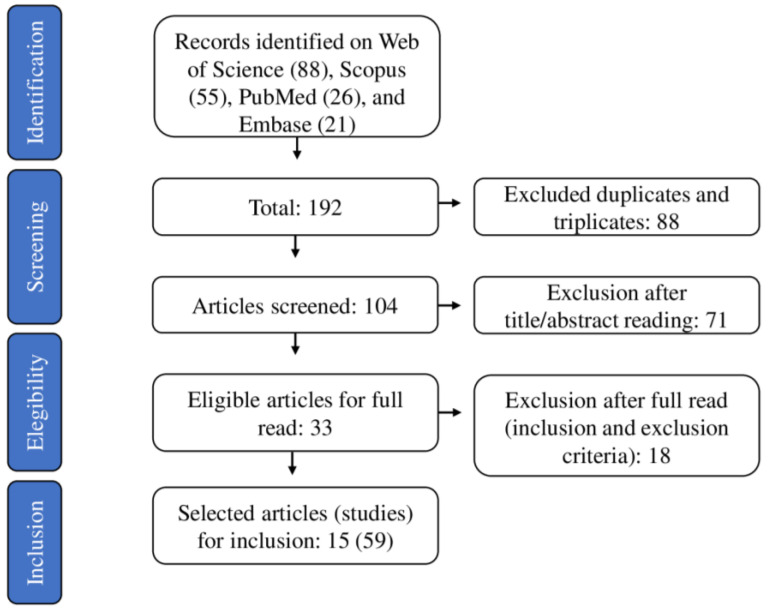
PRISMA flowchart for the search, selection, and inclusion of the studies.

**Figure 2:**
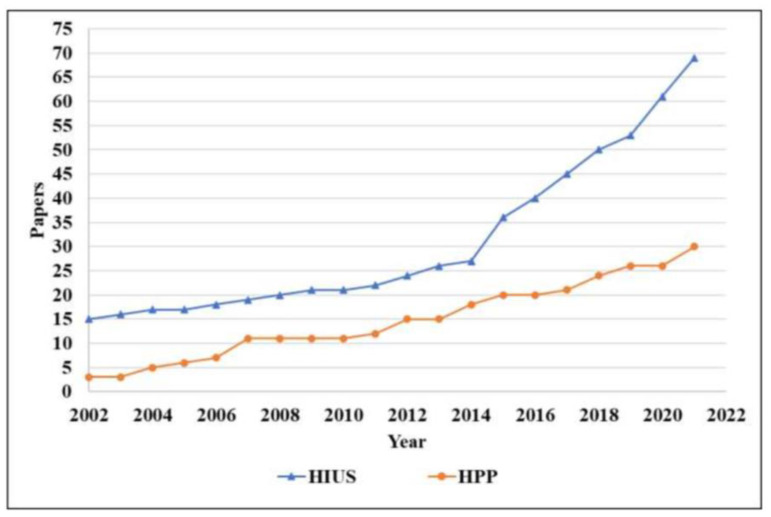
Temporal evolution (last 20 years) of articles on non-thermal technologies’ application in meat tenderization before screening and inclusion steps. HIUS: high-intensity ultrasound, HPP: high-pressure processing.

Figure 1 presents the PRISMA schematic flowchart of the elected articles. A group of 88 articles was identified on Web of Science, 55 on Scopus, 26 on PubMed, and 21 on Embase totaling 192 articles. The 88 related duplicates or triplicates were removed during the screening stage, and 104 papers were maintained. The 104 articles were grouped considering the tenderization technology and the year of publication (Figure 2). These 104 articles were evaluated by reading titles and abstracts, and only 33 were considered eligible for this study, being selected for the full read. Between these, 18 papers were excluded, considering the exclusion criteria. Therefore, 15 papers (59 studies) matched the inclusion and exclusion criteria.

The characteristics of the included articles for each non-thermal technology are shown in Table 1 (HIUS) and Table 2 (HPP). For HIUS processing, the authors used different ultrasonic treatment conditions, including bath or probe HIUS and times varying from 2 to 80 min, in different muscle groups to evaluate shear force, totaling 34 studies to be statistically analyzed, as briefly represented in Table 1. Among the muscle group evaluation, *L. thoracis et lumborum* (LTL) sonication was addressed by 10 studies. Muscle *L. lumborum* (LL) was identified in five studies, whereas *Infraspinatus* (IS) and *Cleidoocciptalis* (CO) sonication was verified by three studies each. *Semitendinosus* (ST) and *Semimembranosus* (SM) were sonicated in 10 and three studies, respectively. For HPP, the applied conditions also varied among the articles (Table 2), including the muscle group (*Biceps femoris* = 3, *Semitendinosus* = 5, *L. thoracic et lumborum* = 4, *L. lumborum* = 2, and thigh muscle = 11), pressure (50–500 MPa), and time (4–30 min) used for treatment, totaling 25 studies.

Additionally, as observed in the included studies, the authors did not limit meat evaluation to shear force and texture profile analyses to describe the changes in meat after sonication or high-pressure processing. Other approaches were applied to better understand physicochemical, microstructural, and sensory alterations that occurred in the matrix, including storage evaluation. Moreover, some studies used combined methods, such as papain enzyme addition in the processing, to improve the tenderization effect. Therefore, the results and discussion of this systematic review were organized into five subsections, comprising: (i) HIUS mechanisms and effects, (ii) HPP mechanisms and effects, (iii) change in the meat matrix after processing, (iv) use of papain combined with NTT as an adjuvant for tenderization, and (v) gaps in the application of HIUS and HPP as tenderization technologies, as disposed of below.

### 3.2. High-Intensity Ultrasound

#### 3.2.1. Tenderization Mechanism and Associated Factors

HIUS is an emerging non-thermal technology that applies acoustic energy in a liquid medium [36]. Ultrasonic waves are transmitted through the liquid medium, forming microbubbles that grow due to oscillatory irregularities (expansion and compression movements) generated by the successive sonication cycles until reaching a critical limit, leading to their implosion [37]. The microbubble implosion releases microjets and generates intense localized heat (5000 K), leading to the pyrolysis of water and the development of free hydroxyl radicals, whose formation is intensified according to the applied power [38]. This phenomenon is known as the cavitation mechanism, which has been widely studied and employed mainly for pathogens reduction and improving food product shelf-life [39]. However, several other applications have been discussed and developed based on the use of HIUS (20–100 kHz, 1–1000 W/cm^2^) in recent years, such as marinating, drying, reducing allergenic proteins, crystallization, homogenization, desalting, and tenderization [37,38,40]. The application of HIUS has drawn special consideration due to the increased demand for green-processed products with lower potential environmental damage [12]. The most recurrent hypothesis of sonochemical tenderization is that HIUS-induced cavitation promotes disruption of cellular membranes, increasing Ca^2+^ (from the sarcoplasmic reticulum) availability for activating the calpain system, and improving the meat tenderization process [41].

The factors of sonication time and treatment intensity present a direct relationship with the acoustic energy density (AED), the proper amount of dissipated energy that occurs during the collapse of the microbubbles, the reason for the main physical and chemical effects caused by cavitation. Thus, it is possible to state that the longer the sonication time, the greater the amount of energy dissipated in the medium, resulting in significant physicochemical changes.

In our study, the overall effect obtained by the meta-analysis was significantly positive in favor of sonication as a meat tenderization technology (SMD = −1.09, CI: −1.69; −0.49, *p* < 0.05), as depicted in Figure S1. The measured heterogeneity was considerable (*I*^2^ = 86%), which can be explained mainly due to the different muscles and animal breeds identified in the included studies (Table 1). Additionally, due to a large number of HIUS-related studies (*n* = 34), we categorize it into three subgroups according to the sonicated muscle (*Longissimus*, *Semimembranosus* and *Semitendinosus*, and *Infraspinatus* and *Cleidooccipitalis*), and into three subgroups according to the sonication time (2–20, 30–40, and 50–80 min), to better estimate the influence of meat composition and time in this processing.

#### 3.2.2. Muscle Composition

Among the studies included in this analysis, five different muscles were used as the matrix for HIUS treatment, as presented in Table 1. However, at least two of the six evaluated muscles (*Semimembranosus* and *Semitendinosus*) had an inconclusive result for the sonication tenderization effect. *Semitendinosus* (ST) is a large muscle that exhibits a high proportion of type IIB fibers [42]. Therefore, the inconclusive result could be related to fiber composition. Muscle fiber composition is a key factor that aids in predicting biochemical changes and meat quality since the metabolism levels in the *postmortem* stage depend on the relative quantity of fiber type that constitutes the muscle [43]. Type I fibers are recognized as slow-twitch fibers, characterized by their increased aerobic capacity and low glycolytic capacity, containing high levels of intracellular lipids and myoglobin compared to type IIB fibers (fast-twitch). Type IIA fibers, on the other hand, would be an intermediary between these two [44].

Also, Listrat et al. [45] pointed out that the total collagen, insoluble collagen, and intramolecular crosslink contents were negatively correlated with SM muscle tenderization. *Postmortem* changes that contribute to meat aging (i.e., proteolysis) are responsible for the breakdown of intramuscular connective tissues (IMCT), composed mainly of collagen fibers [46]. However, this degradation does not seem to be effective in reducing the strength of the remaining IMCT after cooking the meat at temperatures of 70–80 °C (prior step to the shear force analysis) so that the adhesion force between the muscles and fascicles doesn’t be affected by the aging process, as IMCT that contributes to the texture in cooked meat promotes a kind of residual hardness, known as “background toughness” [47]. Therefore, it is possible to hypothesize that this phenomenon is responsible for an apparent improved mechanical resistance of this muscle to the sonication effects.

Moreover, the divalent intramolecular covalent crosslinks formed immediately after the synthesis of collagen fibrils by fibroblasts also play a key role in meat texture. These crosslinks can undergo condensation reactions over the time they remain in the animal’s body, originating the mature crosslinks. In older animals, these mature crosslinks are related to reduced collagen solubility upon heating and, consequently, hardening of the meat [47]. Among the 13 studies included in this analysis, all of them presented were extracted muscle from the carcass of young bulls aged between 20 and 30 months, which does not allow us to state that the inconclusive result is related to the maturation of intramolecular crosslinks [20,22,25,26]. Therefore, it is correct to state that the abundance of type IIB fibers and the IMCT-rich structure would be limiting factors for the HIUS cavitation action, representing a physical barrier against possible damage caused by tissue penetration.

#### 3.2.3. Sonication Time

Figure S2 shows the results of the meta-analysis performed for the effect of different sonication times on meat tenderization. The 34 included studies were divided into three subgroups (2–20 min, 30–40 min, 50–80 min). Sonication presented a beneficial effect on tenderization depending on the time range. The range between 2–20 min (SMD = −0.84, CI: −1.57; −0.12, *p* < 0.05) presented a minor positive effect. This phenomenon could be justified due to the lower AED to which the samples were subjected. On the other hand, the range between 50–80 min (SMD = −4.29, CI: −5.68; −2.91, *p* < 0.05) showed the major beneficial effect. This behavior can be explained by the reduction of total collagen measured in the samples in response to the sonication time, as well as an induced proteolysis activation [41]. Furthermore, the studies in this range (50–80 min) applied only bath sonication for meat processing, which indirectly affects the product [48]. Therefore, it can be hypothesized that bath HIUS could be employed at higher sonication times without generating inconclusive or toughening effects. However, due to the longer processing time to achieve the tenderization, it could be considered a limitation, being an economic disadvantage when compared to probe HIUS.

Surprisingly, in the 30–40 min range, the tenderization effect was measured as inconclusive (SMD = −0.55, CI: −2.10; 1.00, *p* < 0.05), which is contrary to the expected trend. This result could be explained by the majority use of tough muscles (SM or ST) by the included studies in this range and by the oxidative stress environment formed in the matrix. Besides the cavitation mechanism, the Fenton reaction, as recently reviewed by Domínguez et al. [49], could also be correlated to the meat tenderization process. The releasing of hydroxyl free radicals leads meat to an oxidative stress condition, as in a Fenton reaction system, the oxidative rates tend to increase [50]. Protein oxidation could result in the formation of crosslinks, affecting meat structure and spatial arrangement and promoting a toughening effect [51].

Gonzalez-Gonzalez et al. [16] reported that muscles subject to sonication times above 60 min had lower total collagen content than those treated by 60 and 40 min. Higher AED values could be responsible for an improved denaturation of the insoluble collagen content in the muscle, contributing to its tenderization. Therefore, it is hypothesized that as long the treatment time, the greater the tenderization effect of HIUS. On the other hand, sonication for 60 min negatively affects lipid oxidation levels during storage [23]. The hydroxyl free radicals released from the cavitation mechanism could interact with the fatty acids released from fat and fat-like molecules during the aging process, justifying the higher oxidation levels [23]. Therefore, there is a time-related limitation in the application of HIUS and a scientific gap in the optimum sonication time. Moreover, despite the HIUS tenderization has been established in the evaluated ranges, the best time range for treatment application seems to be between 2–20 min, in which the major effect was observed, and undesirable physicochemical changes are close to the minimal.

### 3.3. High-Pressure Processing

#### 3.3.1. Tenderization Mechanism and Associated Factors

HPP is also an emerging non-thermal technology whose principle is to submit packaged food (vacuum packaging) to the pressure in a vessel containing water (or other pressure-transmitting liquid). A hydraulic pump or a piston (indirect and direct systems, respectively) produces the pressure uniformly transmitted through the water to the meat [52]. This process generally occurs at 100–600 MPa, during 3–7 min [53].

According to the Le Chatelier principle, applying pressure favors reactions in which the equilibrium is reached in lower volumes [54]. In general, its use minimally affects the primary structure of proteins since pressure increases do not affect covalent bonds [15]. Additionally, hydrogen bond development and ion disruption are stabilized, reducing volume [55]. Electrostatic and hydrophobic bonds, characteristic of tertiary structure, are also modified, related to hydration changes, leading to protein denaturation and volume reduction [55]. Therefore, HPP treatment could alter proteins’ secondary, tertiary, and quaternary structure, changing their conformation, inactivating enzymes, disrupting cell membranes, and altering internal arrangement without affecting small molecules [15]. High pressure could damage the sarcoplasmic reticulum membranes due to a physical increase in cytosolic calcium concentrations [56]. Besides the activation of the calpain systems, this phenomenon is also responsible for promoting intense muscular contraction, accelerating the process of glycolysis and consequent postmortem changes [56]. These effects are accountable for causing myofibrillar structure breakage and tenderization improvement.

In this study, the overall result indicated a significatively positive effect of high pressure in meat tenderization (SMD = −1.36, CI: −2.2; −0.53, *p* < 0.05), as depicted in Figure S3. The heterogeneity was considerable (*I*^2^ = 88%), similar to HIUS, due to the different muscle groups and animal breeds included in the meta-analysis (Table 2). The studies (*n* = 25) were categorized into four subgroups according to the pressure to understand better the influence of this key variable in the processing.

#### 3.3.2. Pressure Range

The results of the meta-analysis performed for the effect of different pressure conditions on meat tenderization are shown in Figure S4. The 25 included studies were divided into four subgroups (50–200 MPa, 250 MPa, 300–400 MPa, and 450–500 MPa). Pressurization presented a beneficial effect on tenderization depending on the pressure range. Despite the application of HPP having presented an overall positive result for meat tenderization (Figure S3) when categorized into different pressure ranges, it was observed that only treatments performed at 250 MPa were beneficial in promoting tenderization. In this range, only seven studies performed by Ma et al. [17] were included, who applied HPP for tenderizing yak (*Bos grunniens*) meat. Interestingly, yak has extremely tough meat, rich in proteins and low lipid content, harming tenderization [57]. On the other hand, higher pressure levels, even when employed in commonly commercialized species (*B. taurus* or *B. indicus*), showed inconclusive results, tending to be hardening at 450–500 MPa. A possible hypothesis explaining these results would be the negative effects of using HPP in high ranges. As well as the sonication time (30–40 min), pressures above 250 MPa would be associated with an oxidative stress effect. As stated by Medina-Meza et al. [58], HPP is able to accelerate lipid oxidation, and there is a critical threshold pressure between 300–500 MPa, in which oxidation is triggered. Therefore, according to the Fenton reaction mechanism, it is hypothesized that an oxidative environment could lead to a toughening effect, as previously explained. Thus, 250 MPa seems to be the most suitable condition to use HPP to achieve a positive tenderization without undesirable physicochemical alterations in the matrix.

#### 3.3.3. Processing Time

Among the studies included herein, the majority applied HPP processing times equal to or below 15 min (Table 2), which difficulted to group the studies in specific ranges, as was the pressure. However, Ma et al. [17] observed that pressure processing times between 15–20 min (at 250 MPa) could be more efficient for yak (*B. grunniens*) meat tenderization. On the other hand, in shorter times (5–10 min), meat presented increased shear force values. At 5–10 min, the meat tenderization mechanisms mediated by HPP do not reach their maximum capacity, including calcium ions releasing, proteolytic enzyme activation, and muscle fiber damage. Surprisingly, at longer processing times (25–30 min), shear force values obtained were similar to those at shorter times. Although the authors did not explain this behavior, it can be hypothesized that, as well as the excessive pressure, the excessive processing time could be related to the Fenton reaction establishment, generating oxidative-related alterations, and contributing to protein crosslinking and meat hardening. Nevertheless, this is the only paper exploring different time ranges in HPP-assisted meat tenderization. Therefore, further studies should address this hypothesis.

### 3.4. Effects of Tenderization by HIUS and HPP on Meat Quality

Among the complementary analysis performed in the included studies, it could be highlighted physicochemical (pH, instrumental color, water holding capacity, lipid oxidation, and metabolic-derived molecules formation), microstructural (scanning electron microscopy and collagen content and solubility), and sensory changes.

#### 3.4.1. Physicochemical Alterations

The pH of meat subjected to HIUS and HPP treatments tended to increase after processing. This behavior could be related to releasing ions due to these technologies’ mechanical and chemical action and protein denaturation, changing the position of some ionic groups and reducing the exposure of acidic groups of proteins [13,26]. The pH-increasing effect established by both HIUS and HPP is contrary to the immediate pH drops after animal slaughter due to the glycolysis and lactic acid release and accumulation. Therefore, meat treated with NTT is trending to present higher pH values than non-treated ones immediately after NTT application (day 0). This difference tends to increase during storage [16,17]. NTT processing and aging, marked by the improved activity of calpains and other endogenous enzymes, seem to affect meat tenderization significantly. Despite this regard, no studies have previously evaluated and measured if the combined effects of NTT and aging are additive or synergistic, which is currently a key scientific gap in understanding the long-term effects of HIUS and HPP, and differentiating what alterations are individually mediated by these NTT and by aging. Last, Gonzalez-Gonzalez et al. [16] showed pH increases in m. *Cleidoocciptalis* was associated with sonication time, but this behavior was not confirmed for other muscles (*L. lumborum* and *Infraspinatus*). Ma et al. [17] described the same observation on yak meat subjected to HPP processing.

Instrumental color has been extensively evaluated in the studies included herein, mainly by the CIE color coordinates L *, a *, and b *, representing lightness, redness, and yellowness, respectively. Authors described sonicated and pressurized meat with increased L * and decreased a * [16,17,24,30], trending to a light orange color, which is not attractive to the consumers. However, as Carrillo-Lopez et al. [19] stated, color alterations in NTT processed meat are still variable due to differences in the experimental conditions, such as muscle thickness and packaging type. Despite these known barriers to assessing the effects of HIUS and HPP tenderization on meat color, Stadnik and Dolatowski [25] measured the total myoglobin (Mb) content, as well as oxymyoglobin (MbO_2_) and metmyoglobin (MetMb), as the major pigment associated to meat color. The authors suggested that sonication could increase the formation of MetMb immediately after processing, leading to color instability, which could be related to free radicals releasing and increasing oxidation stress. However, no other studies correlate myoglobin fraction content and instrumental color changes.

Water holding capacity (WHC) is crucial during HIUS and HPP tenderization. Short ultrasonic periods appear to increase water-protein interaction, reducing drip loss (DL) and corroborating to avoid meat toughening [59]. The WHC behavior is strictly related to the pH alterations, as previously described, since protein degradation and electrical charges changing (pH increasing), as well as myosin polymerization, leads to a higher WHC [24]. The same trend stated for HIUS was observed by Ma et al. [17] when using HPP on yak meat at pressures between 50–450 MPa. Therefore, both HIUS and HPP could be useful technologies to improve WHC. On the other hand, Duranton et al. [27] described a significant reduction in WHC of pork meat treated by HPP. This reduction was only interrupted when adding salt (NaCl) at 1.5 and 3.0% to the processing. These contrasting results could be explained by the different pressure levels applied by the authors, as higher pressures (500 MPa) seem to establish a negative effect on WHC and, consequently, result in meat toughening. However, to deeply understand the existing gaps in the water-protein mechanisms during NTT processing and meat tenderization, it is necessary to employ more robust approaches, such as low-field nuclear magnetic resonance (LF-NMR), to investigate water population dynamics when meat is subject to these emerging technologies [60].

Meat is a lipid-rich matrix, presenting an increased potential to develop oxidative changes, mainly during storage [49]. The use of sonication for tenderization could improve the formation of reactive oxygen species (ROS) and enhance lipid oxidation product formation, commonly measured as thiobarbituric acid reactive substances (TBARS) [61]. This mechanism was observed by both Garcia-Galicia et al. [21] and Peña-González et al. [23] when applying HIUS to bovine meat tenderization. Despite these negative findings, Garcia-Galicia et al. [21] stated that using a vacuum package is a promising strategy for reducing lipid oxidation levels on sonicated meat, while HIUS intensity was not a significant factor in increasing TBARS values. On the other hand, for HPP, Utama et al. [30] indicated that there is a pressure threshold at 400 MPa (25 °C) in which the formation of ROS as initiators of lipid oxidation occurs, leading to an increase in the TBARS values of HPP-treated meat.

Among the studies included herein, the formation of metabolic-origin molecules, such as ATP breakdown products (e.g., inosinic acid, inosine, and hypoxanthine), total volatile basic nitrogen (TVBN), and volatile organic compounds (VOC) was only evaluated by Utama et al. [30]. Treated fresh meat (500 MPa) exhibits an increase in hypoxanthine and a decrease in both inosinic acid and inosine. Therefore, it is hypothesized that HPP could enhance the activation of phosphatase and nucleoside hydrolase enzymes responsible for converting inosinic acid and inosine to hypoxanthine. On the other hand, vacuum-aged samples presented higher content of inosine. Therefore, it is proposed again that using a vacuum package could extend shelf-life even at elevated treatment pressure conditions, maintaining meat quality. TVBN also increased after HPP tenderization because of bacterial and endogenous protease activity [30]. Additionally, among the VOCs, aldehydes exhibited a significant rise at 500 MPa, which corroborates with the lipid oxidation enhancement attributed to higher pressure levels [30].

#### 3.4.2. Meat Structure and Microstructure

**Figure 3:**
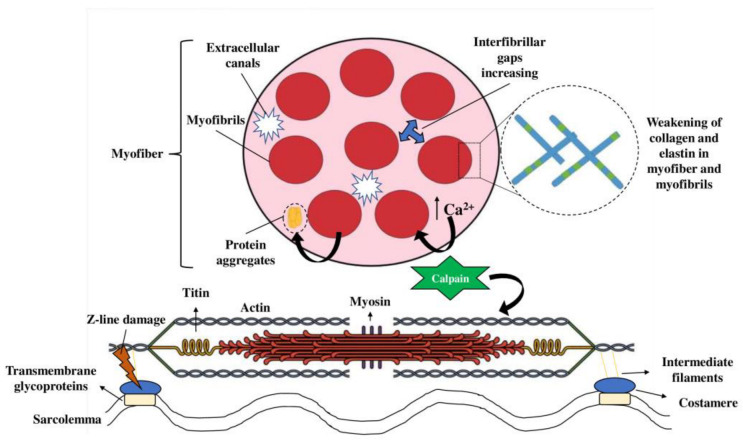
Schematic findings of HIUS and HPP-assisted tenderization effects at the myofibrillar level. Both cavitation and high pressure can cause the weakening of connective tissue and increase the susceptibility of muscle fibers to softening. The action of these NTTs could increase interfibrillar gaps, form extracellular canals, and originate protein aggregates due to myofibrillar degradation. Moreover, the improved releasing of calcium ions, due to mechanical stress, enhance the activation of calpains and other proteases, damaging myofibrillar proteins, such as actin and myosin, and also causing fractures in the Z-line.

Scanning electron microscopy (SEM) and transmission electron microscopy (TEM) has been applied to assess the microstructure profile of different sonicated meat matrices (e.g., beef, pork, and chicken), providing a surface image with resolution down to 0.5 nm and inner structure information, respectively [62]. Therefore, using these imaging techniques in NTT tenderization studies aims to elucidate alterations in the muscle fibers at different levels, from sarcomeres and bands to interfibrillar spaces, as briefly represented in Figure 3.

In general, SEM analysis of sonicated meat describes muscle cell rupture, increased extracellular space, presence of canals, and protein aggregates in the extracellular space [20]. Additionally, sonicated meat could show weakened connective tissue surrounding the muscle fibers [24], which could be explained by the huge number of fractures presented on the Z-line and I-band, as described by Wang et al. [26]. Surprisingly, Carrillo-Lopez et al. [19] found that the increase in the interfibrillar spaces is inversely proportional to the HIUS intensity. In this way, higher intensities could enhance the formation of oxidative initiators, leading to crosslinks and protein aggregation, which is less attractive to promote meat tenderization.

As previously stated in this review, the total collagen content is also a key parameter in assessing HIUS effects on meat tenderization. In this way, Chang et al. [20] described no significant alterations in total collagen content in m. *Semitendinosus* after sonication (1500 W) during 10–60 min. Additionally, the authors suggested that HIUS could not promote disruption of the insoluble collagen crosslinking structure. On the other hand, Gonzalez-Gonzalez et al. [16] verified that the total collagen content changes after sonication are dependent on at least three factors, (i) sonication time, (ii) aging period, and (iii) type of muscle. Collagen-rich muscles, such as m. *Infraspinatus*, which shows high levels of soluble collagen, is more susceptible to HIUS effects. However, sonication effects could only be well characterized after seven days of storage (4 °C), when the longer treatments (e.g., 80 min) result in lower total collagen contents. It should be noted that both studies used similar thickness (2.5 cm) samples [16,20], which highlights the three factors of dependency listed above. In addition, Gonzalez-Gonzalez et al. [16] corroborated the hypothesis Purslow [47] established that the IMCT should be the target when promoting meat tenderization.

#### 3.4.3. Sensory Analysis

Only two articles included in this review performed a sensory evaluation to correlate HIUS’ tenderization with human perception of meat. In general, the texture of sonicated meat was described as soft and juicier than control samples [24]. Therefore, the panelists could perceive the tenderization effect provided by sonication. Moreover, meat was described as pinkish, which corroborates with the L * increased values found in the instrumental color analysis [23]. Meat flavor was enhanced according to the panelists’ perception, which can be explained due the increased release of VOCs, resulting in a product that can be offered to consumers with improved taste and tenderness [24].

### 3.5. Papain: A Good Adjuvant for NTT-Assisted Tenderization?

Papain is a plant-based exogenous enzyme widely used as an artificial tenderization method, being extensively employed in the last years [63,64]. Papain can be obtained from papaya fruit and inactivated only at extreme conditions (900 MPa, 80 °C, 20 min), enabling its application as an adjuvant in NTT-assisted tenderization [65]. This enzyme acts by hydrolyzing large molecules into small peptides and amino acids and could be used on both *antemortem* (injection) and *postmortem* muscle [65]. Despite its recommended dose for meat tenderization being around 0.6% [66], Barekat and Soltanizadeh [18] successfully employed papain at 0.1% combined with HIUS to tenderize m. *L. lumborum*. The findings point that papain activity at 100 and 300 W was not significative different. Furthermore, its combination with HIUS showed promising results, being meat tenderization higher when both methods were simultaneously applied, mainly at 20 min of sonication [18]. Another report on the combined effect of HIUS and papain was recently published by Cao et al. [67], focusing on chicken breast tenderization. Besides the synergistic effect of sonication and the proteases, the authors highlight that combined treatment improved meat WHC. In this way, both HIUS cavitation and enzymatic hydrolysis leads to salt-soluble protein precipitation, enhancing the structural ability to retain water [67]. The combination of papain with HPP was assessed by Ma et al. [17]. At 50 and 250 MPa, the use of papain and HPP simultaneously had no significant differences from the use of HPP alone. However, when using papain (30 min, 55 °C) before HPP (50 MPa, 15 min), the authors observed a higher tenderization effect (46.3%), described as synergistic, suggesting that muscle degradation originated by papain improved HPP capacity to promote tenderization [17]. These findings are promising as they indicate that the use of papain could reduce processing time and energy required by the NTTs, also decreasing potential physicochemical damage. However, to better address meat tenderization and the effect of the combined treatments, further studies on optimization are needed.

### 3.6. HIUS and HPP: How to Fill the Gaps for Industrial Application?

This systematic review and meta-analysis aimed to answer if HIUS and HPP are both NTT available to promote meat tenderization. Despite the overall positive response obtained herein, some points still lack knowledge. Unlike HPP, where the pressure threshold seems to be well established (200–250 MPa), HIUS optimal parameters, such as time and intensity, are not clear yet. Some papers included in the HIUS statistical analysis did not provide data about the intensity of sonication applied, which makes it difficult to elucidate this gap. Additionally, considering the time of treatment and type of equipment, HIUS presents differences. Due to the indirect cavitation exposure, bath sonication is advantageous when considering physicochemical alteration, such as oxidation stress-related ones. On the other hand, it could be considered time-consuming and represent an economic limitation. For HPP, despite the well-determined conditions of pressure and time (generally applied at 5–15 min), there was a large variation in the muscle types used in the included studies. In this way, there was not possible to state if muscle composition influences the HPP efficacy, which was clearly stated to HIUS.

**Figure 4:**
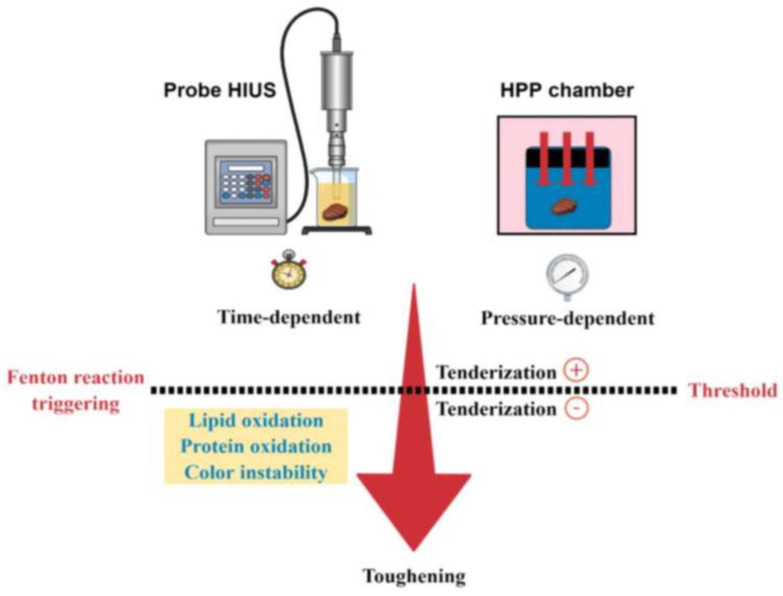
Hypothetical meat tenderization process by HIUS and HPP. Tenderization is part of a general toughening effect, time or pressure-dependent. The threshold between tenderization and toughening is related to the Fenton reaction triggering and the oxidative stress environment.

Moreover, as hypothesized for both HIUS and HPP, the desirable tenderization effect represents the first step of a general toughening effect triggered by these two technologies (Figure 4). The threshold in which the tenderization effect turns from positive to negative seems to be the same as the Fenton reaction starts, enhancing lipid and protein oxidation and crosslinks formation. As described by Estévez [68], protein oxidation directly affects meat tenderization by (i) reducing proteolytic degradation due to the oxidation of histidine and cystine residues in the active sites of calpain, inactivating this enzyme and (ii) strengthening the myofibril structure, via cross-linking. Therefore, to achieve a desirable tenderization, HIUS and HPP processes should be optimized considering the oxidation of main macromolecules (lipids and proteins) that are intrinsically correlated.

Finally, the industrial application of HIUS and HPP should consider using other tenderization approaches, such as exogenous enzymes. Papain has been demonstrating promising results; however, the combination of NTT with other plant-origin (e.g., bromelain and ficin) and microorganism-origin (e.g., *Aspergillus oryzae* and *Bacillus subtilis*) proteases are still lack of knowledge. These enzymes are generally recognized as safe (GRAS) for the meat industry [69] and promote protein breakdown and hydrolysis of collagen and elastin [65], potentializing the effects of HIUS and HPP on tenderization and reducing processing time and costs.

## 4. Conclusions

This study evaluated the applicability of HIUS and HPP as meat tenderization technologies and how intrinsic and extrinsic factors affect this processing. The best sonication time was obtained in the range of 2–20 min, in which the tenderization effect was effective without changing the other physicochemical properties of the meat matrix. The treatment pressure showed better results in the range of 250 MPa, due to the lower release of hydroxyl radicals compared to higher pressures. Muscle composition also influenced HIUS treatment’s effectiveness, where tough muscles, such as SM and ST, proved to be more problematic to softening by sonication due to the higher levels of IMCT content. Moreover, changes in the physicochemical, microstructural, and sensory parameters, mainly focusing on oxidative stress generation, are key factors in achieving desirable tenderization results. Our results suggest that both HIUS and HPP, when used in the optimal application ranges, represent viable alternatives for reaching products with improved softness and, consequently, better consumer acceptability and reduced meat processing time. However, further studies are required to confirm our findings and optimize its use considering the threshold of oxidative damage and combined enzymatic treatments (e.g., papain), expanding its application to different muscles and meat products.

## Figures and Tables

**Table 1:** Detailed description of the HIUS papers included in this meta-analysis.

Breed	Muscle	Conditions	Tenderization ^1^	Findings	Reference
Holstein	*L. lumborum*	Probe, 20 kHz, 100–300 W, 10–30 min	Positive	Lower shear force at 100 W, 20 min; HIUS combined with papain enhanced muscle disruption and proteolytic activity; Myofibrillar swelling due to absorption of water into the myofibrillar spaces.	Barekat & Soltanizadeh [18]
Not informed	*L. thoraci et lumborum*	Bath, 37 kHz, 16–90 W/cm^2^, 20–40 min	Inconclusive	Lower shear force was achieved only after 4 °C storage (7 days); Instrumental color was not significative affected; HIUS modified the myofibrillar structure without changing the shear force; Mesophilic and Enterobacteriaceae counts were controlled during storage.	Carrillo-Lopez et al. [19]
Simmental x Nanyang	*Semitendinosus*	Bath, 40 kHz, 1500 W, 10–60 min	Positive	Lower shear force at 1500 W, 30 min; HIUS had no effects on the insoluble collagen content; Reduction of epimysium and perimysium content by increasing sonication time; Increased sarcomeres shrunk, extracellular spaces, canals, and protein aggregates.	Chang et al. [20]
Angus x Cebu	*Semitendinosus*	Bath, 37 kHz, 16 W/cm^2^ OR 28 W/cm^2^, 40 min. Vacuum package (VP) or modified atmosphere package (MAP)	Negative	Intensity does not significatively affect shear force; Muscles at VP and MAP presented similar shear forces; VP exhibited the potential to inhibit lipid oxidation.	Garcia-Galicia et al. [21]
Brahman x Angus	*L. lumborum*	Bath, 37 kHz, 90 W/cm^2^, 40 min	Inconclusive	HIUS increased water holding capacity (WHC); Improved a * value (redness), as the package is a barrier to pigment extraction.	Garcia-Galicia et al. [22]
Hereford	*L. lumborum, Infraspinatus, and Cleidoocciptalis*	Bath, 40 kHz, 11 W/cm^2^, 40–80 min	Positive	Lower shear force at 11 W/cm^2^, 80 min; Decreasing a * value (redness); Total collagen content does not reduce in muscle *L. lumborum*; Sonicated samples presented higher pH values.	Gonzalez-Gonzalez et al. [16]
Hereford	*L. thoraci et lumborum*	Bath, 40 kHz, 11 W/cm^2^, 60 min	Positive	Lower shear force at 11 W/cm^2^, 60 min; Higher lipid oxidation was observed in treated samples during storage (4 °C, 14 days); Panelists’ perceptions were not altered during the sensory test.	Peña-González et al. [23]
Hereford	*L. thoraci et lumborum*	Bath, 40 kHz, 11 W/cm^2^, 60 min	Positive	Positive effects on flavor, smell, color, and texture after storage (4 °C, 14 days); Wide spaces between muscle fibers and improved degradation of connective tissue.	Peña-Gonzalez et al. [24]
Lowland black-white	*Semimembranosus*	Bath, 45 kHz, 2 W/cm^2^, 2 min	Inconclusive	After four days of storage (4 °C), the shear force of control samples does not differ from sonicated samples; Total myoglobin content does not differ between control and sonicated samples.	Stadnik & Dolatowski [25]
Not informed	*Semitendinosus*	Probe, 20 kHz, 25 W/cm^2^, 20–40 min	Inconclusive	Control and sonicated samples only exhibited significant differences in shear force after three days of storage (5 °C); HIUS improved the activation of μ-calpain, increasing protein breakdown.	Wang et al. [26]

^1^ Tenderization effect was established as positive, inconclusive, or negative, based on the significative or non-significative difference between control and treated samples considering the meta-analysis approach.

**Table 2:** Detailed description of the HPP papers included in this meta-analysis.

Breed	Muscle	Conditions	Tenderization ^1^	Findings	Reference
Not informed	*Biceps femoris*	500 MPa, 6 min, 20 °C	Inconclusive	Treated samples exhibit higher shear force than control samples when cooked; The use of salt (1.5%) prevented the hardening effect of HPP reached at 500 MPa.	Duranton et al. [27]
Holstein	*Semitendinosus*	100–500 MPa, 5 min, 15 °C	Inconclusive	Lower shear force at 300 MPa; Pressure above 300 MPa causes meat hardening; Increased pressure results in higher protein solubility; Calcium ATPase activity decreased at higher pressures.	Y.-J. Kim et al. [28]
Yak	Thigh muscle	50–450 MPa (15 min), 250 MPa (5–30 min)	Positive	Lower shear force at 250 MPa, 15–20 min; Pressure above 250 MPa causes meat hardening; Processing times above 20 min causes meat hardening; WHC was improved at pressures between 50–250 MPa; Treated meat showed higher sensory scores than the control.	Ma et al. [17]
Nelore	*L. thoraci et lumborum*	100–400 MPa, 15 min	Inconclusive	Lower shear force at 200 MPa, with low cooking loss (CL); Decreasing a * value (redness).	Neto et al. [29]
Friesian-Holstein	*L. lumborum*	300 and 500 MPa, 4 min	Inconclusive	Meat treated at 300 and 500 MPa presented no significant differences in shear force; HPP tended to reduce total mesophilic bacteria count and total volatile basic nitrogen (TVBN) content; Reduced inosinic acid but increased hypoxanthine content at day 0; Increased content of volatile organic compounds.	Utama et al. [30]

^1^ Tenderization effect was established as positive, inconclusive, or negative, based on the significative or non-significative difference between control and treated samples considering the meta-analysis approach.

## Data Availability

Data can be requested from authors.

## Supplementary Materials

The following supporting information can be downloaded at: https://www.mdpi.com/article/10.3390/foods12030476/s1, Figure S1: Forest plot of HIUS effect in meat tenderization considering muscle groups as subgroups; Figure S2: Forest plot of HIUS effect in meat tenderization considering sonication time ranges as subgroups; Figure S3: Forest plot of HPP effect in meat tenderization; Figure S4: Forest plot of HPP effect in meat tenderization considering pressure ranges as subgroups.
