# Supplementary material for: Principles, Application, and Gaps of High-Intensity Ultrasound and High-Pressure Processing to Improve Meat Texture
Source: Foods. 2023 Jan 19;12(3):476. doi: 10.3390/foods12030476 (PMC9914770; doi:10.3390/foods12030476)
Supplement: Supplementary file 1 [file foods-12-00476-s001.zip › foods-2139767-supplementary.pdf]

Study or Subgroup	HIUS treated Mean	HIUS treated SD	Control Mean	Control SD	Weight	Std. Mean Difference IV, Random, 95% CI
**Longissimus**						
Barekat et al. (2017)	90	3	96	3	2.5%	-1.60 [-3.82, 0.62]
Barekat et al. (2017)	81	3	96	3	1.3%	-4.00 [-8.18, 0.18]
Barekat et al. (2017)	81	2	96	3	1.1%	-4.71 [-9.53, 0.11]
Barekat et al. (2017)	90	3	96	3	2.5%	-1.60 [-3.82, 0.62]
Barekat et al. (2017)	83	3	96	3	1.5%	-3.47 [-7.18, 0.24]
Barekat et al. (2017)	81	2	96	3	1.1%	-4.71 [-9.53, 0.11]
Carrillo-Lopez et al. (2019)	60.76	1.96	64.68	1.96	2.5%	-1.60 [-3.82, 0.62]
Carrillo-Lopez et al. (2019)	64.68	1.96	64.68	1.96	3.0%	0.00 [-1.60, 1.60]
Carrillo-Lopez et al. (2019)	64.68	1.96	64.68	1.96	3.0%	0.00 [-1.60, 1.60]
Garcia-Galicia et al. (2020)	44	5	39	5	3.2%	0.90 [-0.44, 2.24]
Gonzalez-Gonzalez et al. (2020)	35	1	41.5	5	3.4%	-1.74 [-2.71, -0.78]
Gonzalez-Gonzalez et al. (2020)	35	1	41.5	5	3.4%	-1.74 [-2.71, -0.78]
Gonzalez-Gonzalez et al. (2020)	35	1	41.5	5	3.4%	-1.74 [-2.71, -0.78]
Pena-Gonzalez et al. (2017)	35	1	48	1	1.4%	-12.55 [-16.52, -8.59]
Pena-Gonzalez et al. (2019)	20.41	1.8	22.95	1.8	3.5%	-1.38 [-2.12, -0.64]
**Subtotal (95% CI)**					**36.8%**	**-1.80 [-2.67, -0.94]**
Heterogeneity: Tau² = 1.73; Chi² = 55.44, df = 14 (P < 0.00001); I² = 75%						
Test for overall effect: Z = 4.08 (P < 0.0001)						
**Semimembranosus/Semitendinosus**						
Chang et al. (2015)	56.84	1.96	59.78	3.92	3.5%	-0.92 [-1.68, -0.16]
Chang et al. (2015)	52.92	1.96	59.78	3.92	3.4%	-2.15 [-3.08, -1.23]
Chang et al. (2015)	43.12	2.45	59.78	3.92	3.0%	-4.96 [-6.48, -3.43]
Chang et al. (2015)	49.98	2.94	59.78	3.92	3.4%	-2.75 [-3.79, -1.72]
Chang et al. (2015)	46.06	2.94	59.78	3.92	3.2%	-3.85 [-5.12, -2.59]
Chang et al. (2015)	52.92	2.94	59.78	3.92	3.5%	-1.93 [-2.81, -1.04]
Garcia-Galicia et al. (2019)	32	1	27.5	1.5	3.2%	3.41 [2.08, 4.73]
Garcia-Galicia et al. (2019)	40.5	2.5	34	1	3.2%	3.30 [2.00, 4.59]
Garcia-Galicia et al. (2019)	32.5	1.5	27.5	1.5	3.2%	3.22 [1.94, 4.50]
Garcia-Galicia et al. (2019)	34	1	34	1	3.5%	0.00 [-0.80, 0.80]
Stadnik & Dolatowski (2011)	71	4	69.5	3	3.6%	0.41 [-0.31, 1.14]
Wang et al. (2018)	6.93	0.82	7.12	0.83	3.5%	-0.22 [-1.03, 0.58]
Wang et al. (2018)	6.71	0.68	7.12	0.83	3.5%	-0.52 [-1.34, 0.29]
**Subtotal (95% CI)**					**43.7%**	**-0.54 [-1.65, 0.57]**
Heterogeneity: Tau² = 3.89; Chi² = 208.19, df = 12 (P < 0.00001); I² = 94%						
Test for overall effect: Z = 0.95 (P = 0.34)						
**Infraspinatus/Cleidooccipitalis**						
Gonzalez-Gonzalez et al. (2020)	33	3	38	5	3.0%	-1.05 [-2.62, 0.51]
Gonzalez-Gonzalez et al. (2020)	26.5	5	28	5	3.5%	-0.29 [-1.09, 0.52]
Gonzalez-Gonzalez et al. (2020)	24.5	5	28	5	3.5%	-0.68 [-1.50, 0.15]
Gonzalez-Gonzalez et al. (2020)	22	2	28	5	3.4%	-1.52 [-2.45, -0.59]
Gonzalez-Gonzalez et al. (2020)	38	5	38	5	3.1%	0.00 [-1.39, 1.39]
Gonzalez-Gonzalez et al. (2020)	33	1.5	38	5	3.0%	-1.18 [-2.78, 0.43]
**Subtotal (95% CI)**					**19.5%**	**-0.75 [-1.20, -0.30]**
Heterogeneity: Tau² = 0.03; Chi² = 5.48, df = 5 (P = 0.36); I² = 9%						
Test for overall effect: Z = 3.24 (P = 0.001)						
**Total (95% CI)**					**100.0%**	**-1.09 [-1.69, -0.49]**
Heterogeneity: Tau² = 2.50; Chi² = 282.77, df = 33 (P < 0.00001); I² = 88%						
Test for overall effect: Z = 3.57 (P = 0.0004)						
Test for subgroup differences: Chi² = 4.97, df = 2 (P = 0.08), I² = 59.8%						

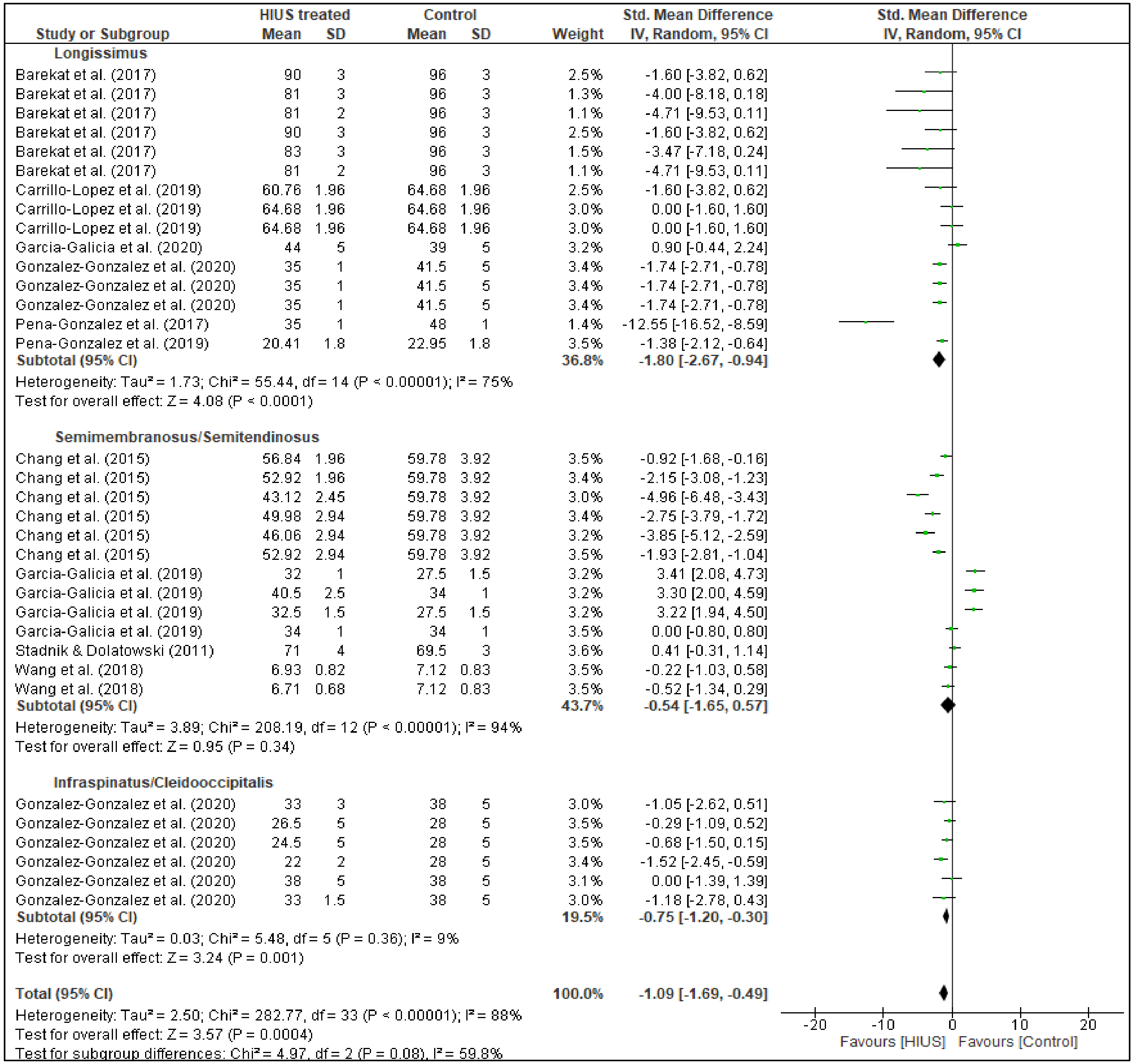

Figure S1. Forest plot of HIUS effect in meat tenderization considering muscle groups as subgroups.

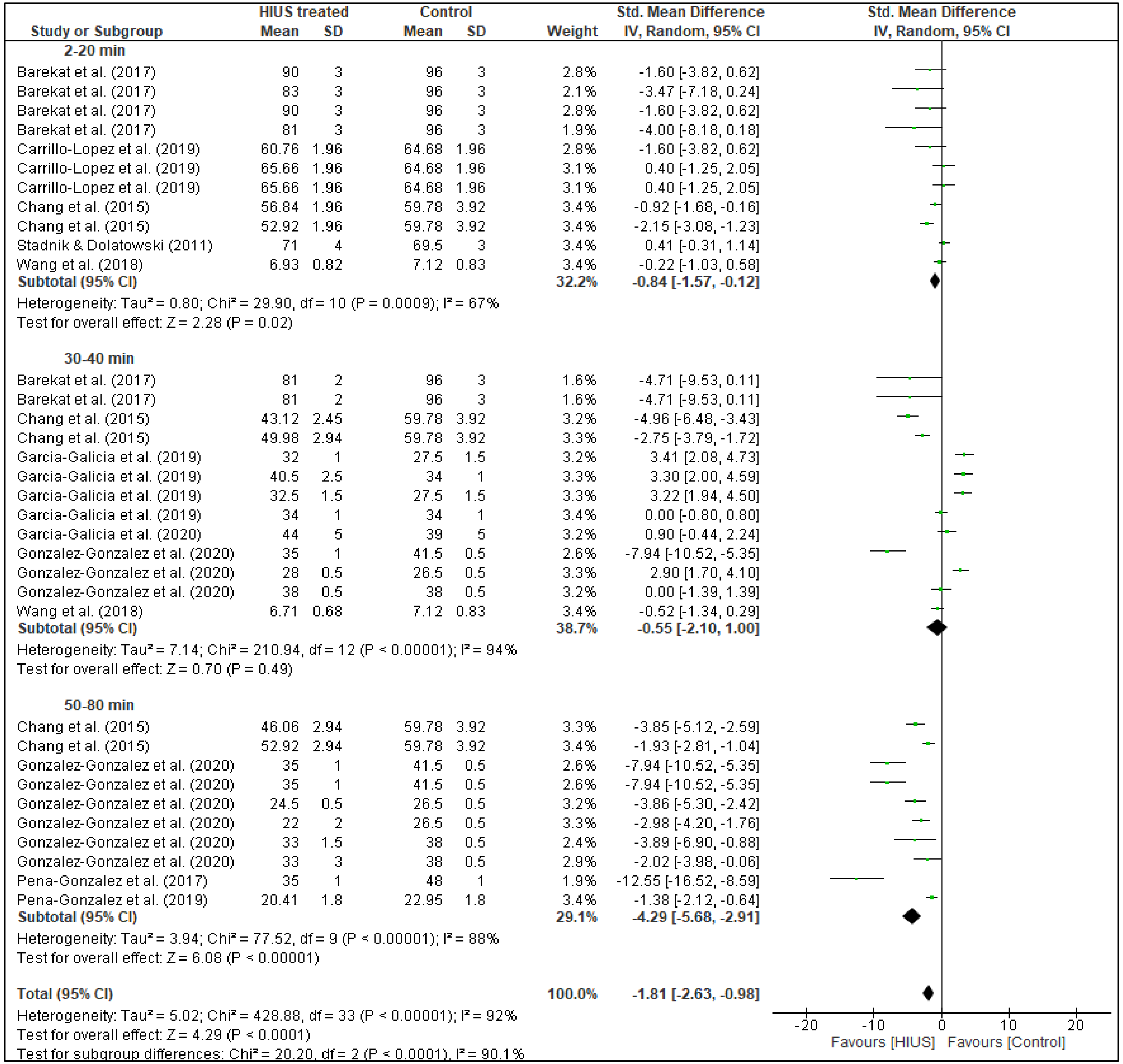

Figure S2. Forest plot of HIUS effect in meat tenderization considering sonication time ranges as subgroups.

Study or Subgroup	HPP treated Mean	HPP treated SD	Control Mean	Control SD	Weight	Std. Mean Difference IV, Random, 95% CI
Duranton et al. (2012)	90	15	56	5	4.9%	2.96 [1.88, 4.03]
Duranton et al. (2012)	50	11	50	4	5.2%	0.00 [-0.72, 0.72]
Duranton et al. (2012)	40	10	42	15	5.2%	-0.15 [-0.87, 0.56]
Kim et al. (2007)	80.066	10.094	73.01	15.19	4.8%	0.49 [-0.78, 1.76]
Kim et al. (2007)	69.874	7.644	73.01	15.19	4.8%	-0.24 [-1.48, 1.01]
Kim et al. (2007)	57.918	9.8	73.01	15.19	4.7%	-1.07 [-2.44, 0.31]
Kim et al. (2007)	73.108	12.74	73.01	15.19	4.8%	0.01 [-1.23, 1.25]
Kim et al. (2007)	98.196	14.602	73.01	15.19	4.6%	1.53 [0.02, 3.04]
Ma et al. (2019)	85	15	132	2	3.8%	-4.05 [-6.33, -1.77]
Ma et al. (2019)	78	4	132	2	1.0%	-15.76 [-23.54, -7.99]
Ma et al. (2019)	70	1	132	2	0.2%	-36.20 [-53.90, -18.49]
Ma et al. (2019)	110	5	132	2	3.3%	-5.33 [-8.17, -2.49]
Ma et al. (2019)	122	8	132	2	4.7%	-1.58 [-2.95, -0.21]
Ma et al. (2019)	102	2	140	10	3.5%	-4.86 [-7.49, -2.23]
Ma et al. (2019)	100	1	140	10	3.4%	-5.20 [-7.97, -2.42]
Ma et al. (2019)	72	1	140	10	2.1%	-8.83 [-13.29, -4.37]
Ma et al. (2019)	72	10	140	10	3.0%	-6.28 [-9.54, -3.01]
Ma et al. (2019)	90	1	140	10	2.9%	-6.49 [-9.86, -3.13]
Ma et al. (2019)	112	10	140	10	4.4%	-2.58 [-4.28, -0.89]
Neto et al. (2015)	20.69	2.75	22.75	3.24	4.8%	-0.63 [-1.81, 0.54]
Neto et al. (2015)	19.81	1.86	22.75	3.24	4.8%	-1.03 [-2.27, 0.21]
Neto et al. (2015)	25.11	1.37	22.75	3.24	4.8%	0.88 [-0.33, 2.09]
Neto et al. (2015)	28.05	0.98	22.75	3.24	4.6%	2.04 [0.54, 3.55]
Utama et al. (2018)	50.764	5	47.138	5	5.0%	0.69 [-0.33, 1.70]
Utama et al. (2018)	50.47	5	47.138	5	5.0%	0.63 [-0.38, 1.64]
**Total (95% CI)**					**100.0%**	**-1.36 [-2.20, -0.53]**

Heterogeneity: $Tau^2 = 3.43$; $Chi^2 = 192.43$, df = 24 ($P < 0.00001$); $I^2 = 88\%$
Test for overall effect: Z = 3.19 (P = 0.001)

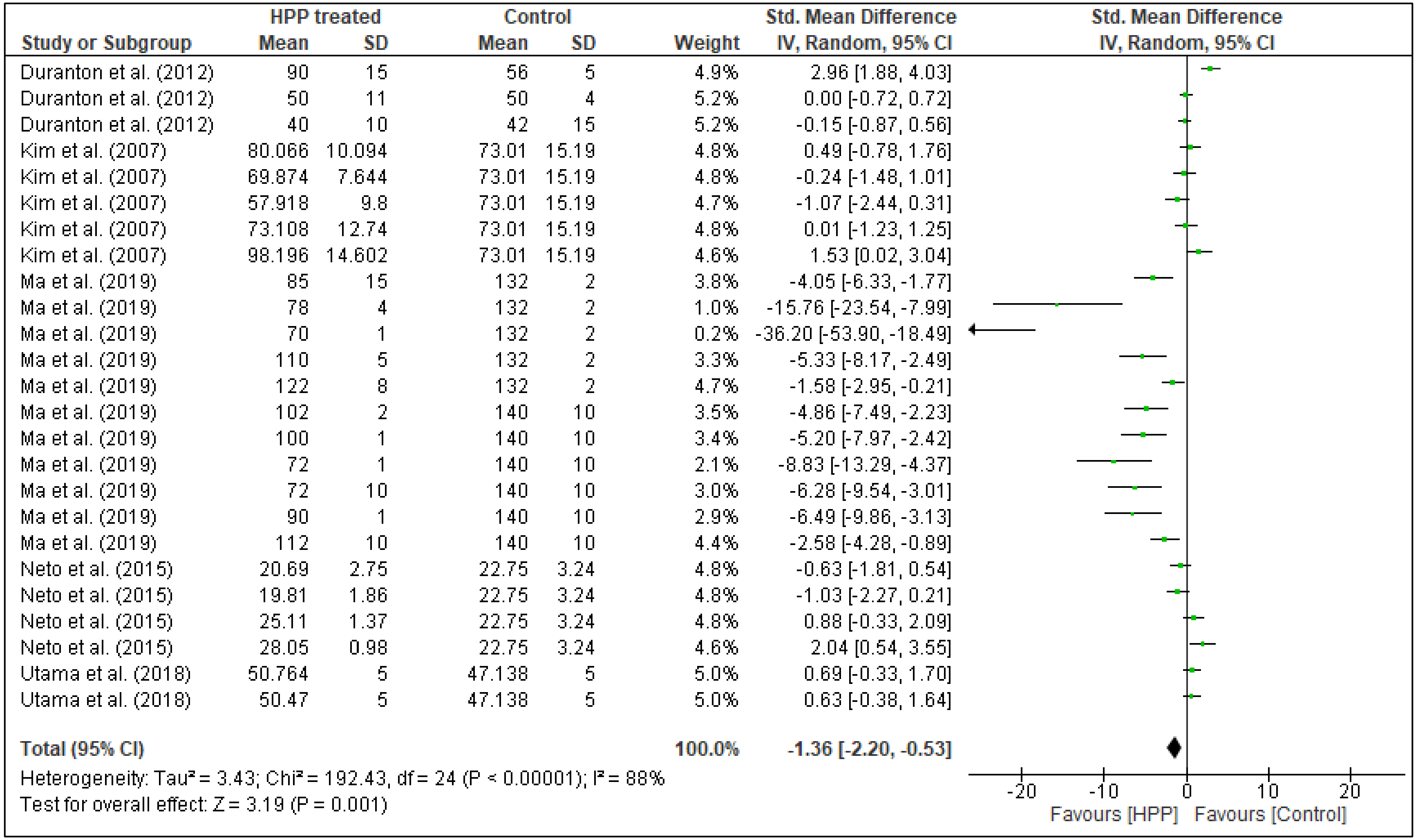

Figure S3. Forest plot of HPP effect in meat tenderization.

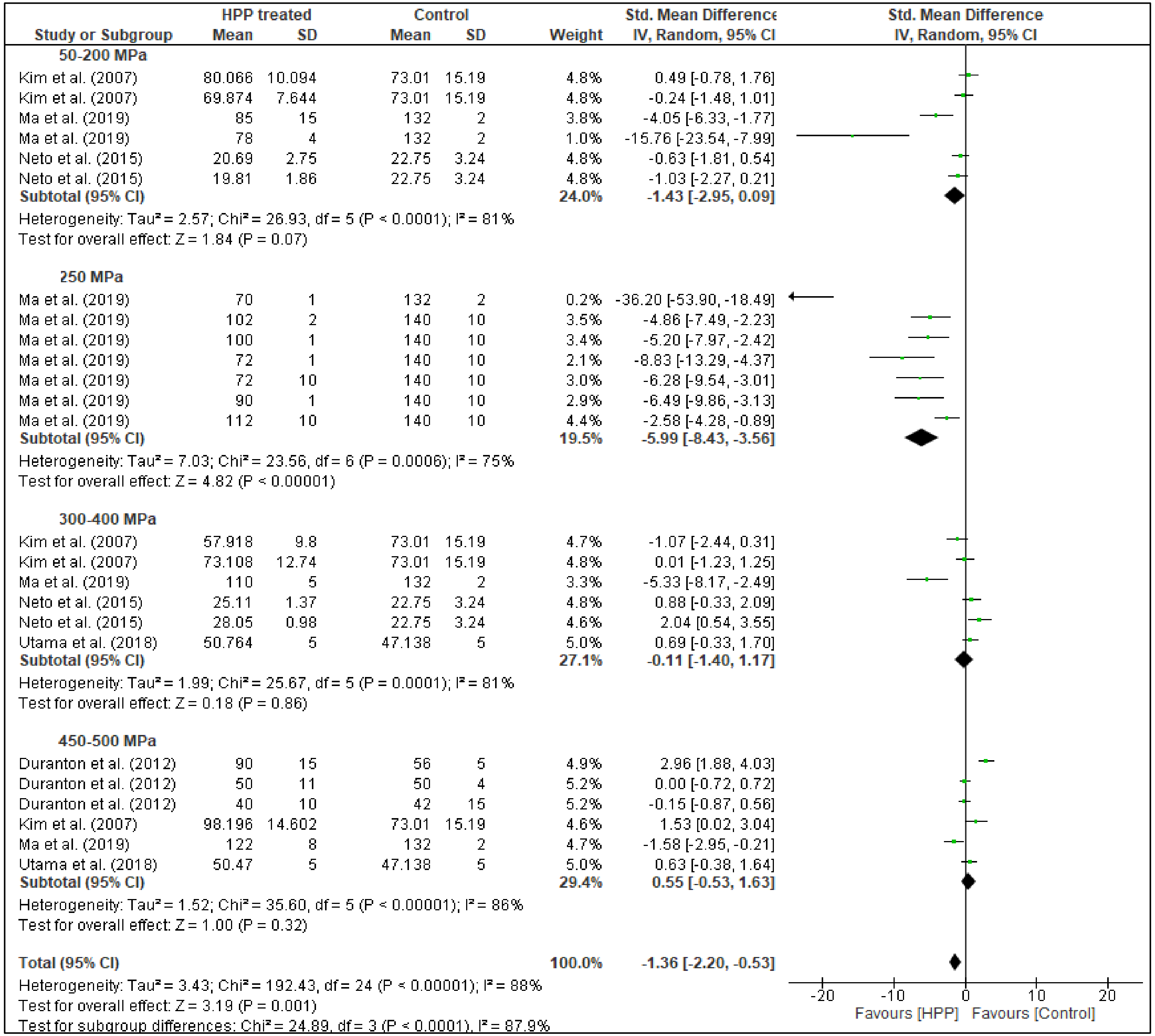

Figure S4. Forest plot of HPP effect in meat tenderization considering pressure ranges as subgroups.
